# Biocompatible MgFeCO_3_ Layered Double Hydroxide (LDH) for Bone Regeneration—Low-Temperature Processing through Cold Sintering and Freeze-Casting

**DOI:** 10.3390/bioengineering10060734

**Published:** 2023-06-19

**Authors:** Hyoung-Jun Kim, Prescillia Lagarrigue, Jae-Min Oh, Jérémy Soulié, Fabrice Salles, Sophie Cazalbou, Christophe Drouet

**Affiliations:** 1 CIRIMAT, Université de Toulouse, CNRS, Toulouse INP, 31030 Toulouse, Francechristophe.drouet@cirimat.fr (C.D.); 2 Research Institute, National Cancer Center, Goyang 10408, Republic of Korea; 3 Department of Energy and Materials Engineering, Dongguk University, Seoul 04620, Republic of Korea; 4 Institute Charles Gerhardt des Matériaux (ICGM), Université de Montpellier, CNRS, ENSCM, 34090 Montpellier, France

**Keywords:** LDH, porosity, 3D bone scaffolds, cold sintering, freeze-casting, computational chemistry, drug delivery

## Abstract

Layered Double Hydroxides (LDHs) are inorganic compounds of relevance to various domains, where their surface reactivity and/or intercalation capacities can be advantageously exploited for the retention/release of ionic and molecular species. In this study, we have explored specifically the applicability in the field of bone regeneration of one LDH composition, denoted “MgFeCO_3_”, of which components are already present in vivo, so as to convey a biocompatibility character. The propensity to be used as a bone substitute depends, however, on their ability to allow the fabrication of 3D constructs able to be implanted in bone sites. In this work, we display two appealing approaches for the processing of MgFeCO_3_ LDH particles to prepare (*i*) porous 3D scaffolds by freeze-casting, involving an alginate biopolymeric matrix, and (*ii*) pure MgFeCO_3_ LDH monoliths by Spark Plasma Sintering (SPS) at low temperature. We then explored the capacity of such LDH particles or monoliths to interact quantitatively with molecular moieties/drugs in view of their local release. The experimental data were complemented by computational chemistry calculations (Monte Carlo) to examine in more detail the mineral–organic interactions at play. Finally, preliminary in vitro tests on osteoblastic MG63 cells confirmed the high biocompatible character of this LDH composition. It was confirmed that (*i*) thermodynamically metastable LDH could be successfully consolidated into a monolith through SPS, (*ii*) the LDH particles could be incorporated into a polymer matrix through freeze casting, and (*iii*) the LDH in the consolidated monolith could incorporate and release drug molecules in a controlled manner. In other words, our results indicate that the MgFeCO_3_ LDH (pyroaurite structure) may be seen as a new promising compound for the setup of bone substitute biomaterials with tailorable drug delivery capacity, including for personalized medicine.

## 1. Introduction

The increased aging of the population worldwide, allied with recurrent traumas and skeletal diseases, generates a high need for osteosynthesis materials (screws, plates…) and bone substitutes [1]. Progress in medical devices and biomaterials research has allowed, in the last decades, great development in terms of types of biomaterials, shapes, porous structures, and physical–mechanical and chemical properties so as to address diverse clinical needs (e.g., whether for load-bearing or non-load-bearing sites), including for personalized medicine [2]. An essential requirement regards the biocompatibility of the envisioned compounds. There is also a need for multifunctional bone substitutes, exerting not only osteogenic properties but also other functionalities, such as antibacterial or anti-inflammatory, so as to optimize and accelerate bone healing and prevent postoperative complications such as infections [3]. Indeed, bone surgeries—whether in orthopedics, dentistry, cranial or maxillofacial domains—are often invasive operations associated with high risks of infiltration of pathogenic microorganisms. In addition, post-surgical pain and sometimes high inflammatory response should also be addressed when needed from a clinical viewpoint. Such additional features can be provided upon doping the implanted biomaterials with bioactive ions (to a non-cytotoxic level) and/or a combination with drugs in view of the local release of active agents at the implantation site. Ions such as Mg^2+^ or Sr^2+^, for example, are well-known osteogenic cations capable of promoting the activity of bone cells to favor tissue healing and new bone formation [4,5]. Among antibacterial agents, antibiotics are ubiquitously utilized via systemic administration to prevent or treat infections, but bacterial resistance phenomena incite the development of systems allowing for local delivery [6] at the surgical site where the action is needed so as to limit the overall antibiotic dose and better focus the antibacterial effect.

Among mineral compounds able to carry and release either ions or molecular species are layered double hydroxides (LDHs). They exhibit a lamellar structure involving metal hydroxide-based layers and anions/water intercalated in the interlayer spaces. As such, they may be seen as “anionic clays” [7]. Their generic composition can be written as [(M^2+^)_1−x_(M^3+^)_x_(OH)_2_]^x+^[(A^m−^)_x/m_(H_2_O)_n_]^x−^, but they are commonly abbreviated on the basis of the nature of the M^2+^, M^3+^, and A^m−^ ions, in the form M^2+^M^3+^A^m−^ LDH. Common examples are MgAlCO_3_ or MgAlCl LDHs, crystallizing in the hydrotalcite structure. With LDHs, two types of interaction with surrounding ions or molecules may come into play: (*i*) surface adsorption and (*ii*) intercalation between adjacent layers. Indeed, not only does the surface of LDH (nano)crystals offer an extended 2D environment propitious for molecular sorption, but the layered structuration itself can also be exploited to transport ions or molecular substances [8]. The partition of the doping ions or combined molecules between the surface and the interlayer spaces is, however, highly dependent on the chemical nature of the LDH considered—exhibiting a more or less stable structure and specific surface characteristics (charge, surface energy…)—but also on the ions or molecular species to be combined. This is ultimately driven by the thermodynamics of equilibria of sorption and intercalation, especially when considering anionic entities that may exhibit a non-negligible affinity for the interlayer spaces of the LDH structure so as to potentially displace the initially intercalated anions (CO_3_^2−^, Cl^−^…) [9,10,11,12].

This dual adsorption/intercalation ability of LDHs has thus been exploited for years in different applicative domains, including depollution and medicine. However, often, the considered LDH chemical compositions did not specifically include biocompatible ions. In particular, Al^3+^ ions are frequently encountered in the stable LDH structures, but the implication of these ions in neurodegenerative diseases such as Alzheimer’s remains debated [13,14]. Nonetheless, in the field of drug delivery, previous illustrations of LDH doping with active agents such as antibiotics or anti-inflammatory drugs have been reported [15,16,17]. Yet, although LDHs appear very relevant for drug delivery purposes, the vast majority of applications developed so far in biomedicine were based on suspensions of dispersed LDH particles. Thin films [18], nanosheets [19], and aero- or xerogels [20,21] have been examined, but the lack of specific mechanical cohesion has limited their practical application. Recently [22], we unveiled for the first time—essentially based on the study of the MgAlCO_3_ LDH (hydrotalcite structure) of different particles sizes—that cold sintering was an original way to obtain cohesive 3D monoliths while preserving some access to interlayer spaces (likely through lateral openings of the lamellar structure). This discovery now opens the way to the preparation of different types of consolidated LDH monoliths for applications where some degree of mechanical cohesion is necessary, as in bone repair. Finally, it may be interesting to explore also ways to obtain porous 3D scaffolds involving LDH particles to enlarge the variety of self-supported constructs for bone regeneration.

In this context, we have focused in the present work on a biocompatible LDH composition involving only ions naturally present in the organism, namely MgFeCO_3_, in view of bone applications in non-load-bearing sites. We present here two approaches leading to 3D constructs involving this LDH compound: (*i*) through the preparation of polymer-based porous scaffolds with embedded LDH particles and (*ii*) by the fabrication of cohesive monoliths by cold sintering (using Spark Plasma Sintering at low temperature). We also investigate the ability of this MgFeCO_3_ LDH compound to be loaded with active molecular agents in view of local release on the basis of experimental results complemented by computational chemistry calculations. Finally, we verified the biocompatibility of the MgFeCO_3_ composition on osteoblastic cells.

## 2. Materials and Methods

### 2.1. MgFeCO_3_ LDH Synthesis

The MgFeCO_3_ LDH powder was synthesized by titrating an aqueous solution of Mg(NO_3_)_2_∙6H_2_O (0.25 M) and Fe(NO_3_)_3_∙9H_2_O (0.125 M) with an alkaline carbonated solution (1 M NaOH/0.3 M NaHCO_3_) up to pH 9.5. Typically, a dropping funnel was set above a round bottom flask containing Mg/Fe mixed metal solution. The alkaline solution was added dropwise to the metal solution while the electronic pH meter was plunged into the solution. Approximately two equivalents of NaOH over metal species were consumed to reach pH 9.5 for 30 min, and the stirring was maintained for 24 h after the titration was over. After the designated time, the suspension was subjected to centrifugation at 8000 rpm for 10 min to retrieve the precipitate, which was further washed thoroughly with deionized water and lyophilized.

### 2.2. Freeze-Casting Processing

Porous 3D scaffolds comprising an alginate polymeric matrix and MgFeCO_3_ LDH embedded particles were prepared by the freeze-casting approach. To this aim, a sodium alginate solution (Sigma–Aldrich, Saint-Quentin-Fallavier, France) was first prepared by dissolution in deionized water at pH 9, with an alginate concentration of 1 wt.%. The alginate used was characterized by size exclusion chromatography (SEC), leading to the following characteristics: number average molar mass (Mn) = 34,620 g.mol^−1^, weight average molar mass (Mw) = 105,000 g.mol^−1^, dispersity (Đ) = 3.03. The LDH powder (0.3 g) was suspended in 15 mL deionized water and mixed with the alginate solution (15 mL), and homogenized using a sonication probe (3 min, 30% intensity, 2 s on/2 s off). The homogenized suspension was then transferred into cylindrical molds (2 cm diameter, 5 cm height) with 4 mL per mold. The latter was covered by copper caps and thermally isolated by placing a peripheral insulating polymer foam. The lower parts of the molds were then connected to a copper cold finger in a homemade freeze-casting device set to −10 °C and left to freeze for 4 h. The molds were then opened, and the frozen suspension was lyophilized for 24 h (Christ Alpha 2-4 LD2, Osterode am Harz, Germany) at −80 °C, 0.05 mbar.

### 2.3. Cold Sintering Processing

Cold sintering was carried out by Spark Plasma Sintering (SPS) at the PNF^2^ CNRS national platform related to the CIRIMAT laboratory in Toulouse, France. The experiments were performed on a Dr. Sinter 1050 equipment (Sumitomo Coal Mining Co., Tokyo, Japan). Uniaxial pressing was applied at 100 MPa (in 1 min) accompanied by indirect heating thanks to a DC pulsed current (max. used in this work of 1000 A), with a 12:2 pulse sequence (12 pulses of 3.3 ms followed by two intervals of 3.3 ms without current). The experiments were created in a vacuum. Heating (100 °C/min) up to 130 °C followed by a plateau for 15 min was used. The monolith was prepared with LDH only (without added components); for each LDH monolith prepared, 0.4 g of powder LDH was introduced in a graphite mold (8 mm internal diameter). 

### 2.4. Physicochemical Characterization

Structural analyses were carried out by X-Ray Diffraction (XRD) on an X-ray diffractometer (AXS D2 Phaser, Bruker, Mannheim, Germany) with a scanning range 2θ = 5–70° using a scan step of 0.02° and the Cu Kα radiation (λ_Cu_ = 1.5406°). Mean crystallite sizes were estimated using Scherrer’s equation applied to diffraction lines (003) and (006) along the c-axis and in a perpendicular direction by following the line (042).

Fourier transform infrared (FTIR) spectra were obtained on a Nicolet iS50 spectrometer in transmission mode (Thermo Scientific, Waltham, MA, USA) in the spectral range 400–4000 cm^−1^ (64 scans, 4 cm^−1^ resolution) using the KBr pellet method. Spectra were analyzed with the OMNIC software (Thermo Scientific, Waltham, MA, USA).

Morphological observations of the starting LDH powder and cold-sintered monoliths were carried out by Field Emission Scanning Electron Microscopy (FE-SEM) using a Quanta 250 FEG microscope (FEI, Hillsboro, OR, USA). For the analyses, the powder samples were suspended in deionized water and drop-casted on a pre-cleaned silicon wafer, while the consolidated LDH monoliths were directly fixed on carbon tape without further treatment. Images were recorded using a 30 kV acceleration voltage. SEM images on the freeze-cast scaffolds were obtained on a Quanta FEI 450 microscope (FEI, Hillsboro, OR, USA) in low vacuum mode at an acceleration tension of 12.5 kV, both on the top of the scaffolds and after the longitudinal cut.

The porous structure of the freeze-cast scaffolds was further examined by X-ray computed microtomography (µCT) using a Phoenix/GE Nanotom 180 instrument (GE Sensing & Inspection Technologies GmbH, Phoenix|X-ray, Wunstorf, Germany) from the French Research Federation FERMAT (FR3089) equipped with a molybdenum target operated at 30 kV/200–250 µA and an HCD-5184-50 detector (2300 × 2300 pixels). Samples were positioned at 35 mm from the X-ray target, and a 35-mm distance was selected between the source and the detector. Images were recorded with a counting time of 1 s/picture and an average of 7 pictures/step. Data processing was made with the Datos X^®^ software (GE Sensing & Inspection Technologies GmbH, Phoenix|X-ray, Wunstorf, Germany) to obtain 3D reconstruction images of the scaffolds. Images (maximum voxel size of 14 μm) were further treated with the Vg Studio Max^®^ software 2.1 (Volume Graphics GmbH, Heidelberg, Germany).

The surface area of the powder and monoliths was quantified by nitrogen (N_2_) adsorption at 77 K using the BET (Brunauer–Emmett–Teller) method. Experiments were run on a Belsorp II mini apparatus (MICROTRAC BEL CROP., Osaka, Japan) after degassing at 120 °C for 12 h. Isotherms of N_2_ adsorption/desorption were analyzed using the BJH (Barrett–Joyner–Halenda) methodology. 

The porosity of the freeze-cast scaffolds was investigated using a mercury intrusion porosimeter (AutoPore III, Micromeritics Instruments Inc., Norcross, GA, USA), allowing for the detection of pores in the range 360–0.003 μm. The overall porous volume was calculated using the equation P_tot_ = d_app_ × V_Hg_ × 100, where d_app_ is the apparent density of the scaffold, and V_Hg_ is the total mercury intrusion volume per gram of specimen analyzed. 

Thermogravimetry and differential thermal analyses (TG/DTA) were made on a Setsys Evolution thermobalance (Setaram/KEP TECHNOLOGIES, Caluire, France). The experiments were run in air, from 30 to 900 °C, at a heating rate of +5 °C/min.

The mechanical behavior of alginate/LDH 3D scaffolds (cylindrical, height ≈ 1.1 cm, diameter ≈ 2.1 cm) was evaluated by uniaxial compression. The tests were performed with an MTS Criterion Model 42 electromechanical system (MTS Systems SAS, Créteil, France) equipped with a 2 kN load cell (acquisition frequency of 1 Hz). A controlled displacement of the upper plate was used to axially compress the samples. (displacement rate during loading and unloading of 0.085 mm/s). An initial preload of 2 N was applied to improve the flatness of the contact surface. The tests were run (up to a compression load of 20 N. The mechanical behavior of consolidated cold-sintered LDH monoliths was evaluated by diametral compression tests (Brazilian tests) as previously reported on disk-shaped specimens. Mean fracture stress values (maximal load to rupture) were measured by pressing each tested monolith (12 mm diameter, 3 mm height) using a Hounsfield press model H25K-S (Hounsfield, Redhill, UK) equipped with a 25 kN force sensor (France Scientifique, St Genis Laval, France). A loading speed of 0.5 mm/min was used.

### 2.5. Incorporation of Molecular Species/Drugs

#### 2.5.1. Interaction with Methyl Orange (MO) as Model Drug

Methyl Orange (MO) was used here as a “model” drug to develop the proof-of-concept of drug incorporation/release from MgFeCO_3_ LDH. In the first stage, the kinetics of retention of MO on MgFeCO_3_ LDH powder or monolith was followed in order to determine the time needed to reach equilibrium. To this aim, the adsorbent (1.3 mg of powder or 1.95 mg of monolith) was added in 1 mL of 2300 ppm (experimental value: 2291 ppm) of MO solution and stored at 37 °C for various time points ranging from 5 min to 24 h (1440 min).

The adsorption isotherm of MO on MgFeCO_3_ LDH was then determined by contacting the LDH powder or monolith (1.3 mg of powder or 1.95 mg of monolith) with 1 mL of each MO solution with equilibrium concentration from 120 ppm to 3500 ppm (3.5 g/L). Then, the LDH powder or monolith in contact with the MO solution was stored at 37 °C for 24 h.

After the adsorption step, MO release from MgFeCO_3_ LDH monolith (MO loading of 1.307 mg/g) was followed in deionized water at 25 °C over 3 h.

In all cases, MO titration in solution was carried out by UV–vis spectrometer (UV-1800, Shimadzu, Kyoto, Japan). To this aim, the supernatants of each sampled solution containing MO were retrieved and filtered with a syringe filter, and the MO concentration was quantified by determining the absorbance at λ = 464 nm by comparison with a calibration line (R^2^ = 0.9953). The adsorbed amount was, in each case, calculated by comparing the initial and final MO concentrations in the solution.

#### 2.5.2. Interaction with Tetracycline (TC) and Ibuprofen (ibu) Drugs

The interaction between MgFeCO_3_ LDH and two actual drugs of clinical relevance, namely tetracycline (TC) and ibuprofen (ibu), was also followed. To this aim, 1 g LDH was contacted with each drug solution, typically at a concentration of 20 g/L, prepared by dissolving 0.4 g of the drug in 20 mL of solvent or equivalent *m*/*v* ratios. The experiments were carried out for 3 days, either at room temperature for TC or at 60 °C for ibu. The interaction isotherm was investigated using a range of concentrations from 5 to 40 g/L. In the case of TC, deionized water was used as a solvent; for ibu—exhibiting a low solubility in water—the dissolution was instead undergone in absolute ethanol.

TC and ibu titrations were undergone by UV–visible spectrophotometry with a Shimadzu 1800 (Shimadzu, Kyoto, Japan) apparatus at a wavelength of λ = 430 nm for TC (secondary peak beside the most intense 355 nm peak to limit saturation) and λ = 264 nm for ibu. Calibration lines were obtained from a series of standard solutions with increasing concentrations in the same range as above (correlation coefficients R^2^ = 0.9985 for TC and 0.9988 for ibu). Prior to analysis, all samples were diluted by a factor of 10 for TC or 100 for ibu. The data were processed through the UV Probe software (version 2.42, Shimadzu, Kyoto, Japan).

### 2.6. Computational Chemistry

The molecular configuration of tetracycline in the LDH interlayer space was investigated by Monte Carlo simulations using a homemade code. For that purpose, the molecular model of tetracycline was built, and the partial charges were extracted from density functional theory (DFT) calculation with DMol3 (Figure S1). During the calculations, geometry optimization was performed using GGA/PW91 functional and DNP basis set, and all electrons were considered for the core treatment. The tolerances to reach convergence were fixed for energy, maximum force, and maximum force variation at 10^−5^ Ha, 0.02 Ha/Å, and 0.005 Å, respectively. The structure of LDH was taken from the methodology described in the literature [23] and by adapting the chemical composition and the unit cell parameters with the ones obtained experimentally. For the LDH, the partial charges were calculated by the electronegativity equalization method. Regarding Van der Waals interactions, the Lennard–Jones parameters were taken from the universal force field (UFF). A multi-cell formed by 3 × 3 × 2 unit cells for LDH was used for calculations, in agreement with a fixed cut-off value for Lennard–Jones contributions of 12.5 Å. The final parameters were thus a = b = 37.2 Å, c = 46.82 Å, α = β = 90°, and γ = 120°. The parameters of the adsorbate/adsorbent Lennard–Jones interatomic potential were then combined using the Lorentz–Berthelot rule. The summation Ewald technique was applied for the long-range electrostatic interactions. The simulations were performed in order to minimize the system energy by introducing a fixed number of anions in the structure and then by determining the saturation of the interlayer space. During these calculations, the motion of the anions included translation and rotation randomly considered during the equilibration steps. For that purpose, 10^7^ Monte Carlo production steps following 2 × 10^7^ equilibration steps were performed at 300 K to elucidate the distribution of interlayer anions. Further calculations were performed to investigate the interactions of tetracycline with the external surfaces by building a slab with the same parameters.

In order to model the interaction of TC in the presence of alginate, complementary Monte Carlo calculations were performed using a model of alginate. In this case, the latter was built by considering the monomer of the alginate structure. Using a similar strategy to the one used before with TC, we investigated the adsorption of TC and alginate on the external LDH surface. As an alginate unit, two has negative charges in both mannuronic acid and guluronic acid units, two configurations have been tested: considering the alginate and 2 Na^+^ in complement of the TC molecules and CO_3_^2−^, or some alginate ions replacing the CO_3_^2−^ as compensating anions to keep the solid globally neutral. Similar parameters for Monte Carlo simulations were considered, while the force field (charges extracted from DFT calculations and UFF for Lennard–Jones parameters) was implemented in the code.

### 2.7. In Vitro Cell Tests

The cytotoxicity of MgFeCO_3_ LDH powder and monolith was evaluated against a human osteoblast-like MG-63 cell line. The MG-63 cell culture line was purchased from the Korean Cell Line Bank, Seoul, Korea. MG-63 cells were cultured with DMEM medium containing 1% penicillin–streptomycin (Welgene, Gyeongsan, Korea) and 10% FBS (Thermo Fisher Scientific, Waltham, MA, USA) in a 5% CO_2_ incubator at 37 °C. The cells were cultured up to 80% confluency. Then cells were seeded in a 96-well plate with a density of 1.0 × 10^4^ cells per well. After incubating for 24 h, LDH monolith extract (9 mg/mL in complete DMEM medium) or LDH powder suspension (prepared by dispersing LDH powder in complete DMEM medium) with various concentrations from 0.01 μg/mL to 10 μg/mL was contacted with the cells and incubated in 5% CO_2_ incubator at 37 °C for 24 h. Cells cultivated without contact with the samples were used as the control group. Then, the cells were washed with PBS and the WST-1 reagent (F. Hoffmann-La Roche, Ltd., Basel, Switzerland) was added. After additional incubation at 37 °C in the CO_2_ incubator for 4 h, the absorbance was measured at λ = 437 nm using a microplate reader (VARIOSKAN LUX, Thermo Fischer Scientific, Waltham, MA, USA).

## 3. Results and Discussion

**Figure 1:**
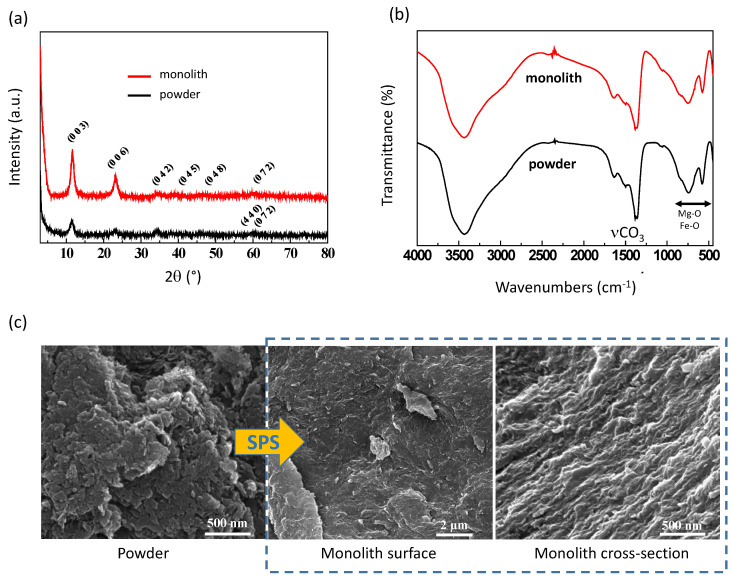
XRD (**a**), FTIR (**b**), and FE-SEM (**c**) results on MgFeCO_3_ LDH before and after cold sintering. The (hkl) index in XRD refers to pyroaurite reference (PDF # 25-521).

In view of developing original bone regeneration applications involving LDHs, we have focused our study on a composition solely involving ions naturally present in vivo, namely Mg^2+^, Fe^3+^, and CO_3_^2−^. Indeed, while iron is present in hemoglobin, magnesium and carbonate ions are present in blood and also, to some extent, in bone tissue [24]. Upon degradation—typically upon osteoclastic resorption—these ions are thus expected to be released and potentially re-used by the organism to fulfill natural functions. The local release of Mg^2+^ ions for example has been reported to favor osteoblast activity to form new bone tissue [5]. This led to the MgFeCO_3_ LDH composition, crystallizing in the pyroaurite structure (space group $R\bar{3}m$), as confirmed by XRD analysis (Figure 1a), corresponding to the chemical formula Mg_6_Fe_2_(OH)_16_CO_3_·4.5H_2_O.

While this compound sounds appealing for bone repair applications of its composition involving only ions naturally present in vivo, it cannot be used as simple powders or suspensions and has to be processed into actual 3D constructs to be used as implantable medical devices. We thus report here our research activities in link with two processing routes, namely freeze-casting and cold sintering, to respectively fabricate (*i*) porous scaffolds involving alginate as a biopolymer matrix and (*ii*) monoliths of densified LDH particles.

### 3.1. Porous 3D Scaffolds by Freeze-Casting

**Figure 2:**
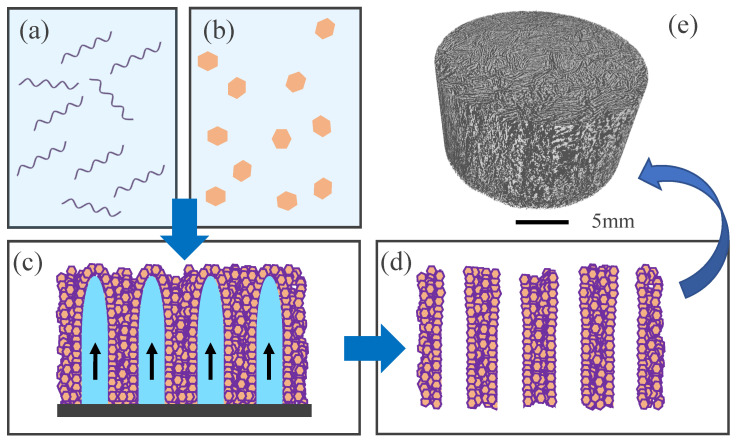
Schematic description of the freeze-casting process: the polymer (alginate in this case) is solubilized (**a**), and inorganic particles (here MgFeCO_3_ LDH) are added (**b**). After homogenization, the suspension is frozen in an oriented way, from bottom to top, to allow the formation of solvent crystals (here water) around which the composite walls are constituted (**c**). Sublimation of the solvent crystals then generates the porosity (**d**). Figure (**e**) is a 3D reconstruction of a typical µCT image: clear areas represent the porosity, while dark areas correspond to the composite polymer/particle walls. Modified from [29].

In this first processing approach, we aimed to prepare 3D porous scaffolds composed of a polymeric matrix with embedded MgFeCO_3_ LDH particles. We used here a well-known resorbable biopolymer of choice in biomedical applications, as in bone engineering, namely alginate (water soluble) [25]. The freeze-casting technology (Figure 2) was then selected as the processing route, as it is particularly well suited to the processing of thermally sensitive materials. In addition, this approach is relevant for bone applications as it allows fabricating of porous scaffolds with oriented, columnar-like porosity favorable for cell colonization, neoangiogenesis, and the percolation of body fluids [26,27,28,29,30].

In this context, we thus prepared 3D alginate scaffolds bearing MgFeCO_3_ LDH particles as mineral charges. This scaffold fabrication path involved two major steps (Figure 2c,d): (*i*) the controlled freezing of the polymer solvent (here water) to generate ice crystals around which the composite walls are constituted and (*ii*) the sublimation of the ice crystals to create the porous network. Taking into account the controlled evolution of the freezing front obtained by our custom-built freeze-casting setup (cold copper finger at the bottom, isolating jacket around the mold), this porous network is expected to be both oriented (e.g., implying rather parallel pore walls) and interconnected (due to micronic defects such as secondary nucleation on LDH particles and supercooled solvent), as shown on Figure 2d,e.

**Figure 3:**
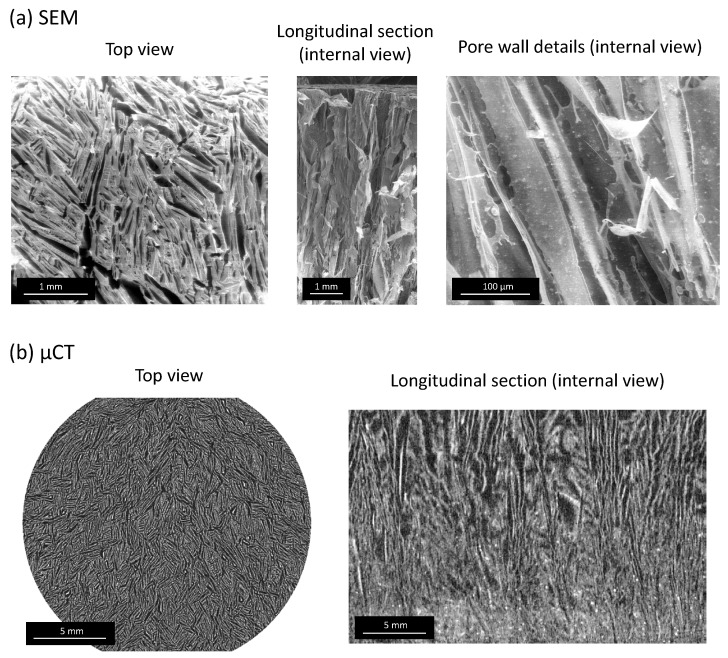
(**a**) SEM micrographs and (**b**) µCT images of freeze-cast alginate/MgFeCO_3_ LDH porous scaffolds.

Typical examples of 3D alginate/LDH scaffolds obtained in this work are shown in Figure 2e and Figure 3. Observation by SEM allowed us to clearly visualize the open-oriented porous network formed by the polymeric walls, as well as the dissemination of the LDH particles embedded in/on the pore walls of alginate (Figure 3a). Complementary microtomography (µCT) analyses run on entire scaffolds confirmed that the expected 3D porous architecture was indeed obtained, evidencing bottom-up parallel-like pore walls throughout the scaffold (Figure 3b). The cumulated porous volume has been measured by mercury intrusion porosimetry, leading to 85.9%.

The mechanical properties of the fabricated scaffolds were followed by uniaxial compression. The stress-strain curves obtained (Figure S2) showed two main modifications by comparing the mechanical behavior of the pure alginate scaffolds (prepared in exact same conditions but without LDH particles) to the composite alginate/LDH scaffolds. First, the initial slope of the curves shows a clear increase in the presence of LDH charges, which can be directly linked to an increase in elastic Young modulus: from σ = 0.02 ± 0.01 MPa for pure alginate to 0.13 ± 0.01 MPa in the presence of LDH particles. Second, this is accompanied by a slight decrease in maximal deformation, from ε_max_ = 1.9 ± 0.2 for pure alginate scaffolds to 1.4 ± 0.1 for the composite scaffolds. Together, these data show that the presence of the inorganic charges (MgFeCO_3_ particles) distributed throughout the 3D scaffold provides complementary mechanical stability by blocking the mobility of polymer chains. This increased rigidity for 3D alginate/LDH scaffolds is a prerequisite to manipulating and cutting samples without collapsing the porosity.

Additionally, the MgFeCO_3_ LDH particles can be conferred extra properties by association with (bio)molecules/drugs in view of local release and bioactivity at a cellular or tissular scale. To this aim, the LDH particles suspended in the alginate solution in the preliminary stages of freeze-casting can be preloaded with dedicated molecular species. The molecular loading capacity of MgFeCO_3_ LDH will be addressed in Section 3.3.

### 3.2. Cohesive Monoliths via Cold Sintering

The second processing approach aimed at the obtainment of self-supported cohesive 3D monoliths made of pure MgFeCO_3_ LDH. However, LDHs are hydrated and often nanosized compounds and thus thermally metastable. They cannot withstand high temperatures that would inexorably lead to structural collapse and irreversible degradation. Therefore, usual ceramic processing routes under elevated temperatures cannot be applied. In this view, there is a need to develop non-conventional consolidation processes to limit the maximal temperatures reached and the time spent under temperature/pressure. Previous studies from Drouet et al. have demonstrated since 2006 that cold sintering could be advantageously used to consolidate metastable inorganic compounds, such as biomimetic apatites, or later amorphous calcium phosphates while preserving their nano and hydrated characters [30,31,32,33]. The cold sintering of other types of materials has also been explored subsequently, e.g., [34,35], and has now become a large area of research worldwide. It may indeed help reduce the regular temperatures of sintering and also be applicable to the processing of thermally-unstable compounds for which non-conventional consolidation routes must be developed. The underlying mechanisms explaining the success of such cold sintering approaches have been discussed on several occasions [22,34,35]. They generally suppose the transient localized formation of a liquid phase (hence the relevance of applying it to already hydrated compounds or the need to add an external source) to facilitate the diffusion of species at lower temperatures than usually required.

**Figure 4:**
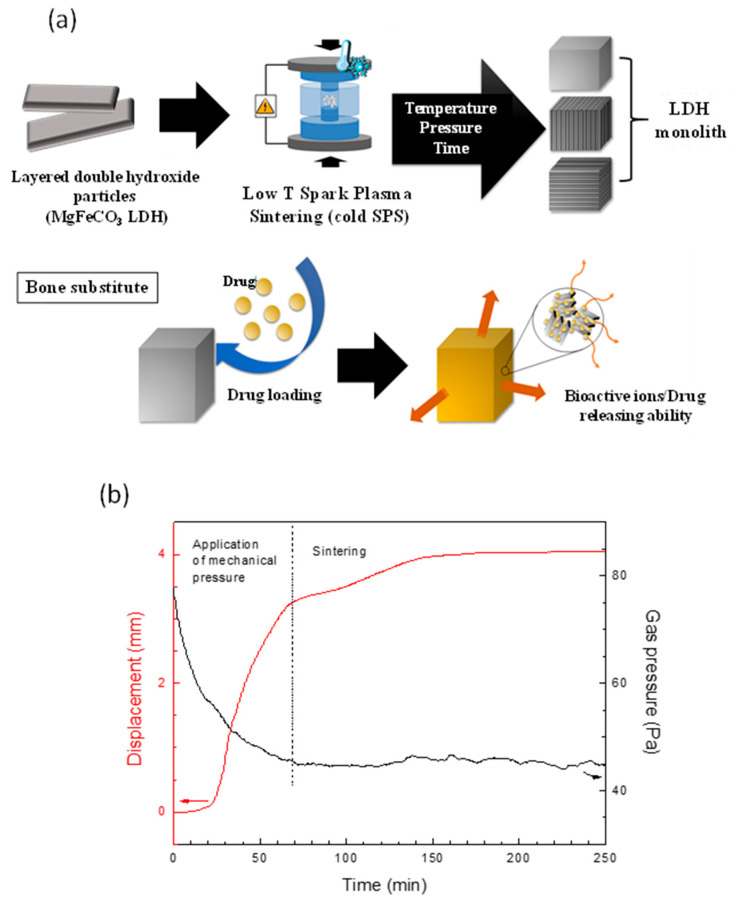
(**a**) General principle of MgFeCO_3_ LDH cold sintering for use as bone substitute eventually loaded with drugs, (**b**) SPS piston displacement and gas pressure follow-up during MgFeCO_3_ LDH cold sintering.

Similar outcomes may thus be expected from cold sintering of LDHs. As indicated in our previous report [22], using cold Spark Plasma Sintering (cold SPS), we indeed showed that MgAlCO_3_ LDH hydrotalcite particles of different sizes could be efficiently consolidated into monolithic pieces, with a more effective sinterability for smaller particles. These findings open the way to novel applications of LDHs in many areas where it is necessary to have solid, self-supported materials, including depollution filters, heterogeneous catalysts, solid electrolytes, filtering systems, etc. We then initiated the investigation of two other LDH compositions, i.e., CaFeCl and MgFeCO_3_, and optimistic outcomes were obtained. In the present work, we aimed to explore the specific preparation of biocompatible MgFeCO_3_ monoliths as bone substitutes and study their association with drugs in view of local release upon biodegradation (Figure 4a).

MgFeCO_3_ LDH monoliths were prepared by cold SPS using the parameters previously optimized for the MgAlCO_3_ LDH. The insulating LDH powder was introduced in a conductive graphite mold, and electrical pulses were applied in combination with uniaxial mechanical pressing (100 MPa) up to reaching 130 °C for a dwell time of 15 min. Cohesive monoliths of homogeneous appearance were then produced. A densification rate of 88% was obtained, with an apparent density of 2.009 ± 0.019 g/cm^3^ (to be compared to the theoretical value of 2.276 g/cm^3^), and the densified state of the LDH sample was evidenced by FE-SEM observations of both the macroscopic surface of the monolith and in the cross-section (Figure 4b). The mechanical strength of such monoliths was evaluated by diametral compression tests, leading to a maximal fracture stress value (maximal load to rupture) of σ = 11.5 ± 3.4 MPa. This is of the same order of magnitude as values obtained by a similar test on nanocrystalline apatites or amorphous calcium phosphates also envisioned for bone repair in non-load-bearing sites [33].

The follow-up of the SPS piston displacement evidenced the existence of an actual sintering step after the first simple compression of the powder upon applying the mechanical pressure, as illustrated in Figure 4b. Interestingly, the gas pressure was also recorded all along the SPS experiments, unveiling the absence of significant release of vapor during the process. This is indicative of the absence of noticeable dehydration/decarbonation of the LDH powder upon consolidation in these conditions, thus allowing us to preserve the composition of the starting powder. XRD and FTIR analyses (Figure 1a,b) concurred to demonstrate the non-alteration of the LDH particles after cold SPS processing, as the initial pyroaurite layered structure was retained with no significant loss of carbonate or major modification. Unit cell parameters and mean crystallite sizes drawn from Scherrer’s equation also remained essentially alike, except for a slight lengthening of the crystallite dimensions along the c-axis, which is probably assignable to the applied pressure/consolidation process (Table 1). We may also remark that the corresponding (003) and (006) XRD lines were more prominent after cold sintering compared to the initial powder (Figure 1a), which can be attributed to preferential orientations as may be expected from compressed layered compounds.

In our previous report on MgAlCO_3_ LDH [22], we showed (using water vapor sorption experiments and computed data on the total accessible surface area) that part of the surface/interlayer spaces of the crystals remained accessible even after consolidation. We can expect that similar conclusions may likely be drawn with the composition investigated here. Specific surface area (S_BET_) measurements via nitrogen adsorption and application of the BET technique showed here a lowering of S_BET_ of about 40% upon consolidation, as expected by the increased density (Table 1). Nonetheless, 1 g of monolith still represents an extended surface area of more than 26 m^2^, thus evidencing a significant developed area. BJH analysis of the nitrogen adsorption/desorption isotherms (Figure S3) allowed evaluation of the porous volume, reaching 0.43 cm^3^/g for the starting powder and 0.30 cm^3^/g for the monolith. Thus, the consolidated MgFeCO_3_ LDH monolith retained nearly 70% of the initial pore volume. However, a clear modification in the adsorption/desorption isotherms shape can be seen between the powder and the monolith, with the formation of hysteresis in the latter case. In the powder, N_2_ adsorption is expected to occur mainly in macropores from inter-particle spaces, which is coherent with the type II/III shape of the isotherm (without significant hysteresis) according to the IUPAC classification [36]. For the cold-sintered monoliths, a change in the shape of mesopores can be noted after consolidation, as evidenced by a large hysteresis. This is indicative of the formation of a tight network between neighboring particles. The hysteresis shape (type H2(b) after IUPAC) then suggests that there is a narrow pore size distribution [36,37], which is coherent with homogenized densification throughout the monolith.

### 3.3. Incorporation of (Model) Drugs

**Figure 5:**
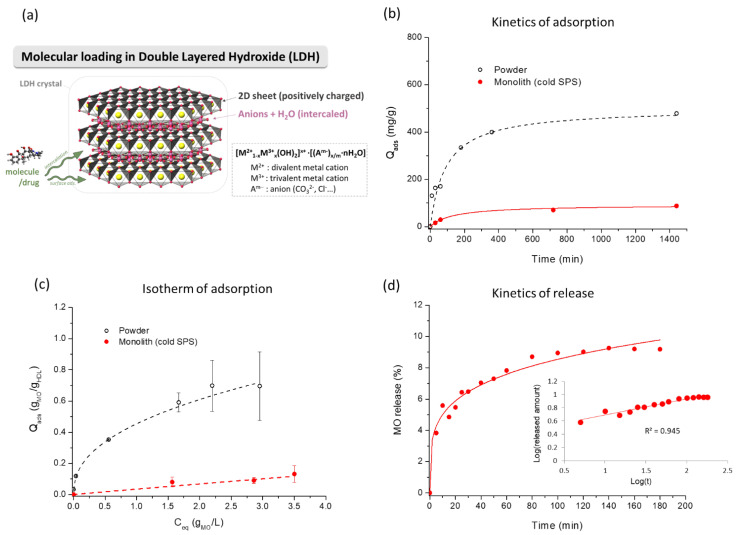
(**a**) Overview of molecular loading in LDHs, (**b**) kinetics of adsorption of methyl orange (MO) as test molecule for MgFeCO_3_ LDH powder and cold-sintered monolith, with pseudo-second-order fit, (**c**) corresponding isotherm of adsorption (at 37 °C) for powder and monolith with Freundlich fit, and (**d**) kinetics of release from the monolith with Korsmeyer–Peppas fit. The inlet in subfigure (**d**) shows the Korsmeyer–Peppas linearization curve.

One relevant approach in the field of biomedical engineering for bone repair relates to the possibility of conveying additional properties by combination with (bio)molecules or drugs in view of local release. In order to develop such a proof-of-concept here, we have first to demonstrate the possible accessibility of crystal surface/intercalation sites to solubilized molecular species (Figure 5a) and as already proven on powdered MgAlCO_3_ LDH [9]. Here, we thus probed in detail about the possible loading in a solution of LDH (as powder or monolith) with a test molecule, Methyl Orange C_14_H_14_N_3_SO_3_^−^,Na^+^ (MO). It should be noted that this molecule was selected as a “model” drug, as it can be easily quantified by UV–vis spectrophotometry, and the negative sulfonate charge is advantageous to interact with the positive surface of MgFeCO_3_.

Figure 5b depicts the typical kinetics of retention of MO (at 37 °C, starting from a concentration of 1.3 g/L for the powder and 1.95 g/L for the monolith. For the sake of simplicity, we will speak of quantity “adsorbed” (Q_ads_), but the term adsorption should be taken in its general meaning, as in the case of LDHs, both internal (interlayer) and external surfaces may be involved in molecular interactions (Figure 5a), depending on the type of LDH and molecules considered, as explicated in the Section 1.

The kinetic follow-up of MO loading in the case of MgFeCO_3_ LDH as powder or monolith reached a stabilized value of around 800 min (~13 h, Figure 5b). The maximal value reached for the monolith was about 5.4 times lower than for the powder (despite a somewhat higher starting concentration), as the densified structure prohibited multilayer adsorption on the surface; yet a significant loading (in this case, ca. 90 mg/g) can be obtained even in the consolidated state. Mathematical fitting of the kinetic curves was achieved by testing the pseudo-first-order and pseudo-second-order models, as is commonly conducted in adsorption kinetic studies. For both the powder and monolith forms, the latter led to better fitting results (Table S1), and the fitted curves can be seen in Figure 5b. Observation of a good agreement with the pseudo-second-order kinetic model for drug adsorption/intercalation of antibiotics in other LDHs (ZnAlCl and Mg/Al nanosheets) has, for example, been reported [15,16], and the authors drew the conclusion that adsorption was limited by the number of accessible sites, suggesting ion-exchange process. Similar conclusions may likely be drawn here, although both external surface sites and intercalation sites may probably come into play. For the sake of completeness, we also analyzed the kinetic data with regard to the Elovich model, which involves a distribution of adsorption energies for different accessible sites [16]. The mathematical fitting process led, in this case, to correlation coefficients R^2^ even slightly larger than for the pseudo-second-order ones (Table S1), reaching, for example, 0.9992 for the monolith. These findings thus globally indicate that quantitative amounts of molecular species can be associated with the MgFeCO_3_ LDH composition at play in this work but also highlight the existence of a variety of sites that can accommodate this test molecule, probably both internal and external, as already proven on powdered MgAlCO_3_ LDH.

Besides the study of the kinetics of adsorption, the determination of the adsorption isotherm (at 37 °C) for a fixed equilibration time of 24 h and varying MO concentrations is also interesting as it allows assessing the “adsorbed” amount versus the concentration at equilibrium C_eq_. As expected, the obtained isotherms (Figure 5c) depict a progressive increase of the amount of drug associated with the LDH particles as C_eq_ increases and in a lower proportion for the monolith compared to the powder. Both isotherms could be satisfactorily fitted to the Freundlich model (R^2^ of 0.9995 for the powder and 0.9755 for the monolith), where the adsorbed amount can be related to the equilibrium concentration by the relation $Q_{ads}=k\cdot C_{eq}{}^{1/n}$. For information, other models such as Sips isotherm [35] led to somewhat lower matches (R^2^ of 0.9912 and 0.9518, respectively). Adequacy of the Freundlich model for molecular associations with some LDHs was also previously reported [16], and, generally speaking, it suggests the existence of different energetically inequivalent sites. These findings are thus in good agreement with the kinetic conclusions drawn above. It may be remarked that the value of the parameter *n* in the Freundlich equation reached 2.35 ± 0.01 for the powder versus 1.04 ± 0.13 for the monolith. On the other hand, the Freundlich constants were *k* = 0.455 ± 0.005 and 0.035 ± 0.006, respectively. The lower values obtained for *k* and *n* in the monolith indicate a lower affinity and sorption capacity than with the powder, which may again be related to the densification of structures and limited multilayer adsorption upon cold sintering. Nonetheless, the increase in Q_ads_ with C_eq_ still depicts a situation where the number of associated molecules can be tailored as a function of the applicative needs—provided that the solubility of the molecule was not a limiting factor to reach the envisioned doping amount.

Since our above results (kinetics and isotherms) point to the possibility of combining molecular species even in the consolidated state, it was also interesting to demonstrate that molecular release was also possible afterward. To this aim, we followed the release of MO from the MgFeCO_3_ monolith after soaking in water initially exempt from MO (Figure 5d). As may be seen, a progressive release of the molecule can then be measured over time. It could be mentioned that the main type of interaction between MO and MgFeCO_3_ LDH must be electrostatic interactions between the MO ions and the layer, and π-stacking interactions among MO molecules, as already observed in MgAlCO_3_ LDH from previous Monte Carlo results [9].

Mathematical modeling of the release curve has been tested versus several popular models, namely zero- and first-order, Higuchi, Hixson–Crowell, and Korsmeyer–Peppas (Figure S4) [38,39]. Only the latter, Korsmeyer–Peppas, led to a reasonably good match with a correlation coefficient of R^2^ = 0.9450 (and being in the validity domain of this model with a maximal release lower than 60% of the total dose). In this model, the fraction of drug released at time *t* is proportional to $t^{n}$ where the value of *n* is informative on the underlying release mechanism. In the present case, it led to *n* = 0.23 ± 0.02. This value lower than 0.5 suggests a Fickian mechanism of release controlled by diffusion. These findings appear rational, taking into account the compressed character of the LDH particles forming a textured monolith as seen by XRD, likely involving intra- and inter-crystal diffusion pathways.

The above findings, therefore, establish the proof-of-concept of loading LDH particles or scaffolds with drugs in view of local release. It is, however, important to keep in mind that the mode of interaction may vary depending on the type of molecular species considered. In order to approach one step further bone applications, the interaction between MgFeCO_3_ LDH particles and two actual drugs of clinical relevance, namely tetracycline (TC) and ibuprofen (ibu), has also been followed. While TC is a well-known wide-spectrum antibiotic of clinical relevance in many domains, including in orthopedics [38,40,41,42], ibu is a common anti-inflammatory drug that may be used in analgesic therapy [43]. The experiments were run in water (at RT) at pH ≈ 7 for TC and in absolute ethanol (at 60 °C) for ibu—hardly soluble in water. For example, Figure S5 reports the illustrative case of the adsorption isotherm obtained after interaction between MgFeCO_3_ LDH powder and TC molecules. As may be seen, the curve obtained exhibits, in this case, the beginning of an S shape isotherm after Giles et al.’s classification [44]. The data points did not allow reaching a plateau in the concentration range explored in this work, 5–40 g/L, thus demonstrating the capacity for TC to interact quantitatively with the MgFeCO_3_ LDH substrate. To the best of our knowledge, this is the first study of the kind on the MgFeCO_3_ compound involving only biocompatible ions. Literature studies on other LDH compositions/structures, often containing Al^3+^ ions, have indicated the possibility of combining antibiotics like tetracycline or oxytetracycline or anti-inflammatory compounds like ibuprofen with LDHs [15,16,17,43], where both external surface interactions and interlayer intercalation may potentially come into play. Here, the interaction isotherm can be fitted (R^2^ = 0.989) with the Sips equation Q = Q_m_K_s_C_eq_^m^/(1 + K_s_C_eq_^m^), as shown by the dotted line in Figure S4. This fit does not allow for the precise determination of Q_m_ as no plateau is yet observed, and thus of K_s_; however, the order of magnitude of the exponent m is less affected by the uncertainty on the (Q_m_, K_s_) values and remains potentially instructive on the type of interaction between adsorbed species. In the present case, its fitted value is m = 2.7 ± 1.5. Despite the rather large uncertainty, this value significantly greater than unity points to positive cooperativity between TC molecules. Again, a good match with the Sips equation suggests rather heterogeneous surfaces for LDH particles with a distribution of interaction sites and the existence of non-negligible interactions between adjacent molecules. Following this curve—or similar ones obtainable in other conditions of temperature, pH, or concentrations—it then becomes possible to tailor the experimental conditions to adjust the drug dosage (TC in this case) to the clinical needs, thus strengthening our concept.

Interaction of ibu with the MgFeCO_3_ LDH powder was more delicate to address, as our observations showed that a parallel phenomenon of partial dissolution of LDH particles occurred in the absolute ethanol at 60 °C for 3 days, leading to significant alteration of the UV–vis spectral features. These conditions were inspired by a previous study on MgAlCl hydrotalcite-like LDH [45]; however, the Fe-based carbonated LDH developed in the present work appeared more sensitive than the Al-based one in our experimental conditions. Adaptation of the methodology could be explored, in future works, to better adjust the time/temperature parameters to be used for MgFeCO_3_ in absolute ethanol. Thermogravimetry (TG, Figure S6) analyses were then used to evaluate the amount of ibu associated with the MgFeCO_3_ LDH powder (1 g) when contacted with an ibu solution at 20 g/L (0.4 g in 20 mL absolute ethanol), leading to a loading of 1.3 mg_ibu_/g_LDH_. In comparison, the loading of TC after contact with an initial TC concentration of 20 g/L is significantly greater and of the order of 0.2–0.3 g_TC_/g_LDH_ as indicated by UV–vis and TG analyses. As expected, the drug loading capacity of the LDH particles largely depends on the nature of the drug. In addition, hydrophobic molecules such as ibuprofen have more difficulty in combining to the polar surfaces of LDH, while hydrophilic drugs like TC may be more easily combined with a tailorable dose dependent on the envisioned application.

**Figure 6:**
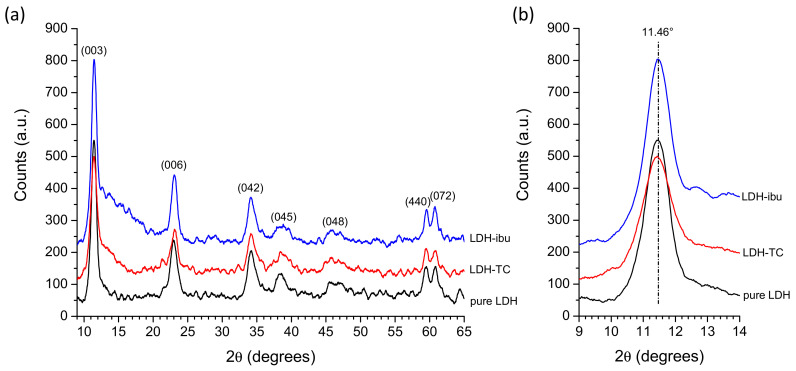
(**a**) XRD patterns of MgFeCO_3_ LDH powder doped or not with TC and ibu, (**b**) zoom on the (003) line in the 2θ = 9–14° range.

XRD analyses were carried out to check the detectable modifications upon interaction with TC or ibu molecules. As shown in Figure 6a, however, no obvious alteration of structural features was noticed compared to the pristine LDH powder. In particular, the position of the (003) line—indicative of the interlayer spacing—did not change noticeably (Figure 6b). These findings suggest either that the intercalated molecules did not significantly modulate the distance between adjacent layers in the LDH structure or that, in our experimental conditions, TC and ibu molecules were essentially adsorbed on the external surface of the LDH crystals. This conclusion could also explain the relatively good fit with the Sips isotherm, as shown in Figure S5.

**Figure 7:**
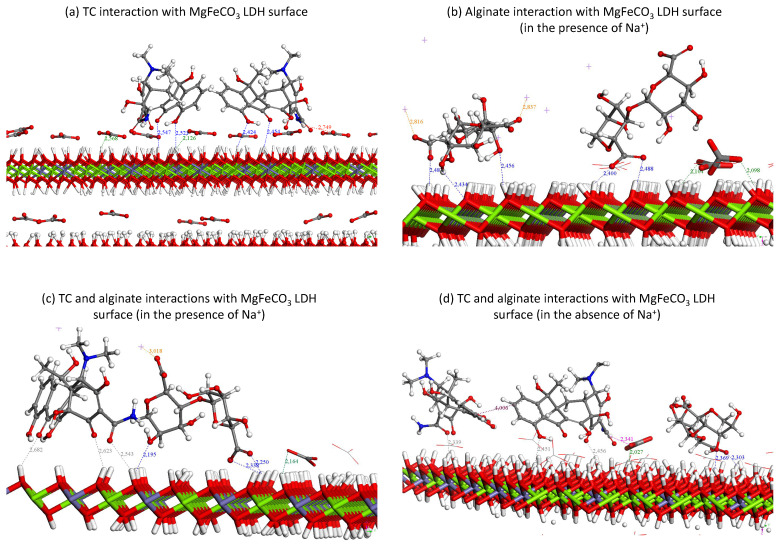
Monte Carlo simulations (built from a slab) displaying stable configurations upon the interaction of tetracycline (TC) or alginate (monomer) with the MgFeCO_3_ LDH external surface: (**a**) interaction with TC, (**b**) interaction with alginate (monomer), (**c**) cumulated interaction of TC and alginate in the presence of sodium counter ions, and (**d**) cumulated interaction of TC and alginate in the absence of sodium counter ions. Sodium ions are shown by the purple cross mark (+). Carbonate ions comprised in the composition of the LDH are also shown. Noticeable electrostatic bonds are indicated with the corresponding bond calculated length.

In order to shed more light on this matter, especially in the case of TC, where an adsorption isotherm could be plotted in our experimental conditions, Monte Carlo calculations have been performed to first investigate the plausible interactions of TC in the LDH interlayer space. The calculations suggest that the (neutral) TC molecule cannot readily enter the structure with the imposed interlayer space opening (from the experimental unit cell parameters listed in Table 1), with CO_3_^2−^ preventing TC entrance due to steric hindrance caused by strong interactions between these divalent anions and the LDH layers. To go further, Monte Carlo simulations have been performed on the slab in order to characterize the possible interactions of TC with the external surfaces. Calculations were performed with 1 and 2 TC molecules in order to investigate the interaction between molecules and LDH surfaces as well as the possible mutual interaction between molecules. Due to the strong interaction between CO_3_^2−^ and LDH surface (with interaction distance ranging from 2.1 to 2.5 Å), TC molecules interact with hydrogen bonds with distances ranging from 2.4 to 2.6 Å. Furthermore, the orientation of the molecules is more or less perpendicular to the layer. Interactions between CO_3_^2−^ and TC can also be observed through hydrogen bonds between -NH_2_ from TC and O from CO_3_^2−^ (with distances ranging from 2.5–2.8 Å). A typical configuration is shown in Figure 7a.

These computational findings thus agree well with our above-reported experimental isotherm and XRD data, suggesting that the essential part of TC molecules is expected to be adsorbed on the external surfaces of the MgFeCO_3_ LDH particles. Yet, quantitative loading through adsorption can be reached, as demonstrated above, with the possibility to modulate the loading dose to adjust to clinical needs.

In this work, interaction with another important organic molecular species, namely the alginate macromolecule, was also considered for the fabrication of freeze-cast porous scaffolds. It was thus also interesting to transpose this simulation methodology to study the interaction between alginate and the surface of MgFeCO_3_ LDH. To run such simulations (while limiting the duration of the calculations), a monomer alginate—along with its sodium counter ions—was considered and introduced in the calculation. Figure 7b reports a typical Monte Carlo configuration. Our results clearly suggest that electrostatic bonding can form between the alginate chain and the LDH layer, implying the –OH or the charged –COO^−^ end groups of the alginate molecule. The length of the bonds (~2.4–2.5 Å) appeared in the same order—somewhat shorter—as those found above for the interaction with TC. It is thus expected that the two molecular moieties can coexist at the surface of MgFeCO_3_ LDH crystals. In our freeze-casting strategy, the alginate is dissolved in water, and LDH particles are added in suspension. TC loading may then be carried out either at this stage, that is prior to the freezing step; otherwise, the LDH particles may instead be preloaded with TC and be added to the alginate solution subsequently. In the former case, there will be a competition between TC and alginate for interaction with the LDH surface. Then, part of the TC drug may ultimately adsorb on the LDH, while another part may remain in the solution and be physically trapped/retained on the polymeric walls forming the porosity of the freeze-cast scaffolds during the sublimation step. Instead, when TC-preloaded LDH is used, the TC drug is expected to essentially remain adsorbed on the LDH particles. Both scenarios could be appealing from a clinical viewpoint, for example, for modulating the release kinetics after implantation. In this case, some end-groups of alginate may access the surface, but TC pre-adsorption is likely to limit this interaction. In all cases, it was interesting to check how computational chemistry could account for the co-adsorption of both molecules. Figure 7c reports a typical configuration after convergence, showing that both molecular species may indeed establish electrostatic bonding with the LDH surface. In this simulation, taking into account the new overall chemical environment and distribution of charges, the calculated bond lengths are slightly shorter for alginate (~2.2–2.3 Å) than for TC (~2.5–2.6 Å). The presence of negatively-charged end-groups on the alginate chain likely conveys a preferential positioning and slightly stronger interaction compared to neutral TC. It may, however, be reminded that, in real experimental conditions, the alginate chain consists of a polymeric repetition of the monomer considered in the calculations of Figure 7 and that actual 3D constraint may convey additional limitations in terms of conformation and interaction with the LDH surface. Nonetheless, these simulations point out the possible electrostatic interaction between the two organic moieties, TC and alginate, even when they are present simultaneously. In order to check the possible secondary role of Na^+^ ions, the same calculation as in Figure 7c was made in the absence of sodium (in this case and to keep a neutral global system, alginate ions replaced some CO_3_^2−^), leading to Figure 7d giving very similar outcomes. Consequently, the Na^+^ ions (counter ions of the alginate precursor) are not expected to generate significant modifications of the organic-inorganic interaction scheme.

### 3.4. In Vitro Biocompatibility Tests

**Figure 8:**
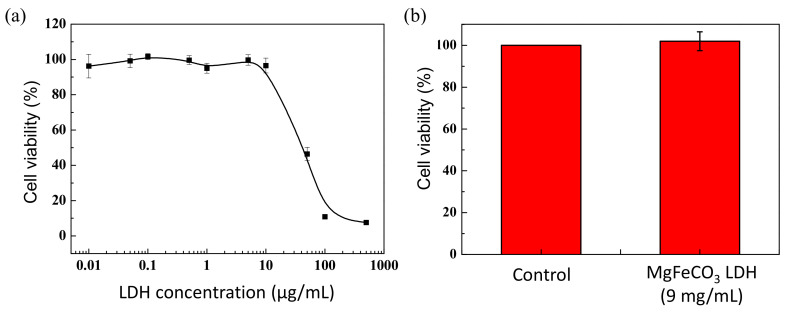
Cytotoxicity evaluation of MgFeCO_3_ LDH against MG-63 osteoblastic cells: (**a**) as LDH powder for increasing LDH concentrations in the medium, and (**b**) as LDH cold-sintered monolith (at the concentration of 9 mg/mL). The control corresponds to the cells alone, without contact with the LDH particles.

As mentioned above, we purposely selected an LDH compound involving only ions that are naturally present in the body to generate a biocompatible composition for use in vivo for bone repair. However, in order to validate this biocompatibility, we ran in vitro tests on osteoblastic MG63 cells. The control group then consisted of the cells alone in the medium, without contact with LDH particles. Although this biocompatibility evaluation is only preliminary, our results validate the good biocompatibility of the MgFeCO_3_ LDH powder toward MG63 cells, as shown in Figure 8a. Indeed, the cell viability remained in the range of 95–100% in a large domain of tested LDH concentrations in the medium, and the start of the decrease only occurred beyond a concentration of ~10 µg/mL (i.e., 10 mg/L).

Since LDH particles can be particularly close to each other in the densified consolidated LDH monoliths prepared by cold-sintering, we also tested a typical monolith of MgFeCO_3_ LDH (at the concentration of 9 mg/mL in the medium), and no decrease in osteoblast cell viability was noticed compared to the control. Again, these results, albeit preliminary, clearly go in favor of good biocompatibility of the LDH composition studied in this work, thus further granting their relevance in the field of bone regeneration.

## 4. Discussion and Concluding Remarks

LDHs are very appealing inorganic (nano)crystalline compounds of relevance to many applicative fields, taking into account their versatility in terms of obtainable chemical composition and their capacity to adsorb and/or intercalate a wealth of ionic and molecular species. However, in the biomedical field, the examples of uses have been for a vast majority addressing nanomedicine applications as in oncology or medical imaging. This may be linked to technical issues for obtaining self-supported 3D constructs capable of being manipulated, cut, and implanted by surgeons as required by clinical needs. In this study, we faced the challenge of preparing bone substitutes with an LDH composition solely involving ions naturally present in vivo, namely Mg^2+^ (an osteogenic ion), carbonate, and Fe^3+^.

The obtained MgFeCO_3_ LDH particles were then investigated as a precursor in the fabrication of 3D pieces to be used as bone substitutes. To this aim, we gather here results on two relevant processing approaches adaptable to metastable compounds such as LDHs (hydrated, nanosized), i.e., freeze-casting and cold sintering. While the first strategy allowed us to obtain highly porous 3D scaffolds based on alginate with embedded MgFeCO_3_ LDH particles, the second approach allowed preparing cohesive textured monoliths. In both cases, mechanical properties were found reasonable for the envisioned applications, keeping in mind the effect of porosity on the mechanical resistance and the objective of implantations in non-load-bearing bone sites. In the alginate-LDH freeze-cast scaffolds, the inorganic particles were found to act as strengthening charges, leading to a clear increase in the scaffold’s Young modulus.

The ability of the MgFeCO_3_ LDH compound to be loaded with molecular moieties was then investigated, confirming that both isolated particles or monolithic LDH pieces were able to take up quantitative amounts in view of local release. The proof-of-concept was created with a model drug, Methyl Orange, and the cases of two actual drugs, namely tetracycline (wide spectrum antibiotic) as well as ibuprofen (anti-inflammatory, analgesic drug), were then examined. Our results pointed out differences in sorption behavior among the three molecular species investigated, as could be expected for molecules exhibiting different charges and hydrophilic/hydrophobic characters. Yet, in all cases, the dose of the drug may be adjusted in a possible range with the aim of meeting the clinical needs. Of particular note is the fact that, even in the consolidated/densified state, the MgFeCO_3_ LDH monoliths could still be “filled” with noticeable loads, highlighting that percolating paths remained accessible, as pointed out by adsorption/desorption isotherms analysis. Computational chemistry calculations, through DFT and Monte Carlo approaches, helped us to examine, with more detail, the type of interaction between LDH particles and two organic moieties relevant to this work (tetracycline and alginate), unveiling the formation of electrostatic bonds in both cases.

We finally checked the cytocompatibility toward osteoblastic cells of this MgFeCO_3_ LDH, either as separated particles (powder) or in the form of densified monoliths, confirming their expected biocompatibility.

This work is expected to illustrate the possibility of fabricating 3D bone substitutes using a fully biocompatible LDH composition, thus initiating the development of a novel family of bioactive bone scaffolds of clinical relevance.

## Figures and Tables

**Table 1:** Comparison of a-axis/c-axis unit cell parameters, crystallite sizes, specific surface areas (S_BET_), and pore volumes (V_P_) for MgFeCO_3_ LDH as starting powder and cold-sintered monolith. Numbers in underscript refer to the next digit.

	Cell Parameters (Å)		Crystallite Sizes (Å)			S_BET_ (m^2^/g)	V_P_ (cm^3^/g)
	a	c	(003)	(006)	(042)		
Powder	12.4	23.4	73.1_2_	53.7_6_	81.1_0_	44.6	0.43_7_
Monolith (SPS)	12.4	23.3	83.9_5_	82.6_4_	72.3_3_	26.3	0.30_4_

## Data Availability

Data are available by contacting the corresponding author(s).

## Supplementary Materials

The following supporting information can be downloaded at: https://www.mdpi.com/article/10.3390/bioengineering10060734/s1: Figure S1: Partial charges obtained for tetracycline and alginate (charged 2-) from DFT calculations. Figure S2: Evaluation of mechanical properties (stress-strain curves) through uniaxial compression for typical freeze-cast alginate/MgFeCO_3_ LDH and pure alginate scaffolds. The zoomed inlet view allows better visualizing of the initial parts of the curves to decipher the variation in Young modulus (slope of the red dotted lines). Figure S3: Evaluation of the porous volume by the BJH method, from processing nitrogen (N_2_) adsorption/desorption data: (a) MgFeCO_3_ LDH powder, (b) SPS cold-sintered monolith. Figure S4: Mathematical fitting of MO release from MgFeCO_3_ LDH monolith (from cold sintering) relative to several models (t is given in minutes and the release rate in %). Figure S5: Isotherm of interaction between TC molecules and MgFeCO_3_ LDH powder. The dotted line depicts the fit with Sips isotherm. Error bars are included but not visible (standard deviations correspond to 0.6% of the mean). Figure S6: Thermogravimetry data on pure MgFeCO_3_ LDH powder, tetracycline (TC), ibuprofen (ibu), and combined LDH-TC and LDH-ibu compounds. Table S1: Correlation coefficient for the mathematical fit of the kinetics of adsorption of MO on MgFeCO_3_ in powder or monolith forms, using the pseudo-first-order, pseudo-second-order, and Elovich kinetic models.
