# Supplementary material for: Biocompatible MgFeCO3 Layered Double Hydroxide (LDH) for Bone Regeneration—Low-Temperature Processing through Cold Sintering and Freeze-Casting
Source: Bioengineering (Basel). 2023 Jun 19;10(6):734. doi: 10.3390/bioengineering10060734 (PMC10294808; doi:10.3390/bioengineering10060734)
Supplement: Supplementary file 1 [file bioengineering-10-00734-s001.zip › bioengineering-2425677-supplementary.pdf]

bioengineering

Supplementary Materials

Biocompatible $MgFeCO_3$ Layered Double Hydroxide (LDH) for Bone Regeneration—Low-Temperature Processing through Cold Sintering and Freeze-Casting

Hyoung-Jun Kim [1,2,3], Prescillia Lagarrigue [1], Jae-Min Oh [2,*], Jérémy Soulié [1,*], Fabrice Salles [4], Sophie Cazalbou [1] and Christophe Drouet [1]

[1] CIRIMAT, Université de Toulouse, CNRS, Toulouse INP, Toulouse 31030, France; christophe.drouet@cirimat.fr (C.D.)
[2] Research Institute, National Cancer Center, Gyeonggi 10408, Republic of Korea
[3] Department of Energy and Materials Engineering, Dongguk University, Seoul 04620, Republic of Korea
[4] Institute Charles Gerhardt des Matériaux (ICGM), Université de Montpellier, CNRS, ENSCM, Montpellier 34090, France; fabrice.salles@umontpellier.fr
* Correspondence: jaemin.oh@dongguk.edu (J.-M.O.), jeremy.soulie@toulouse-inp.fr (J.S.)

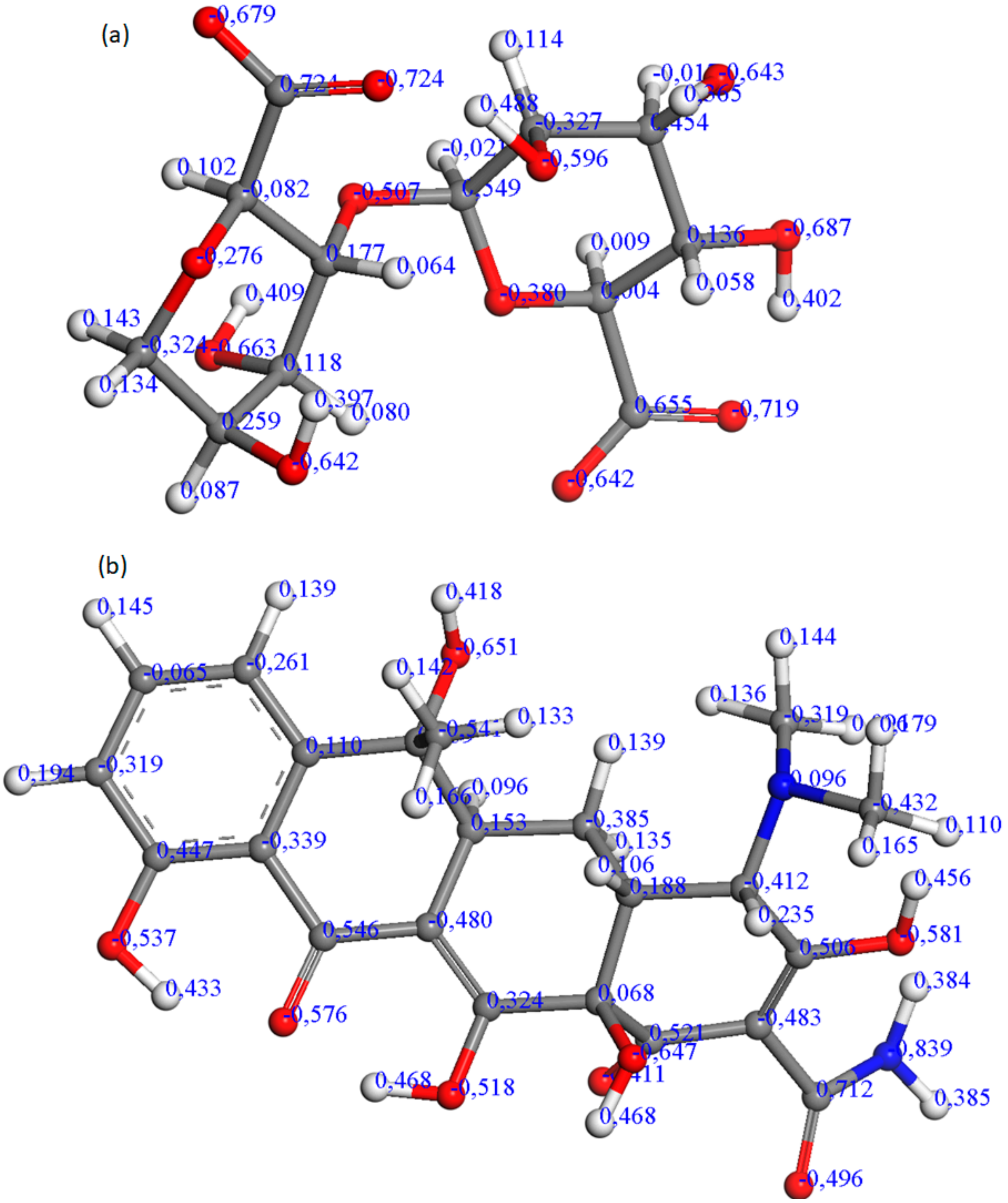

Figure S1. Partial charges obtained for tetracycline and alginate (charged 2-) from DFT calculations.

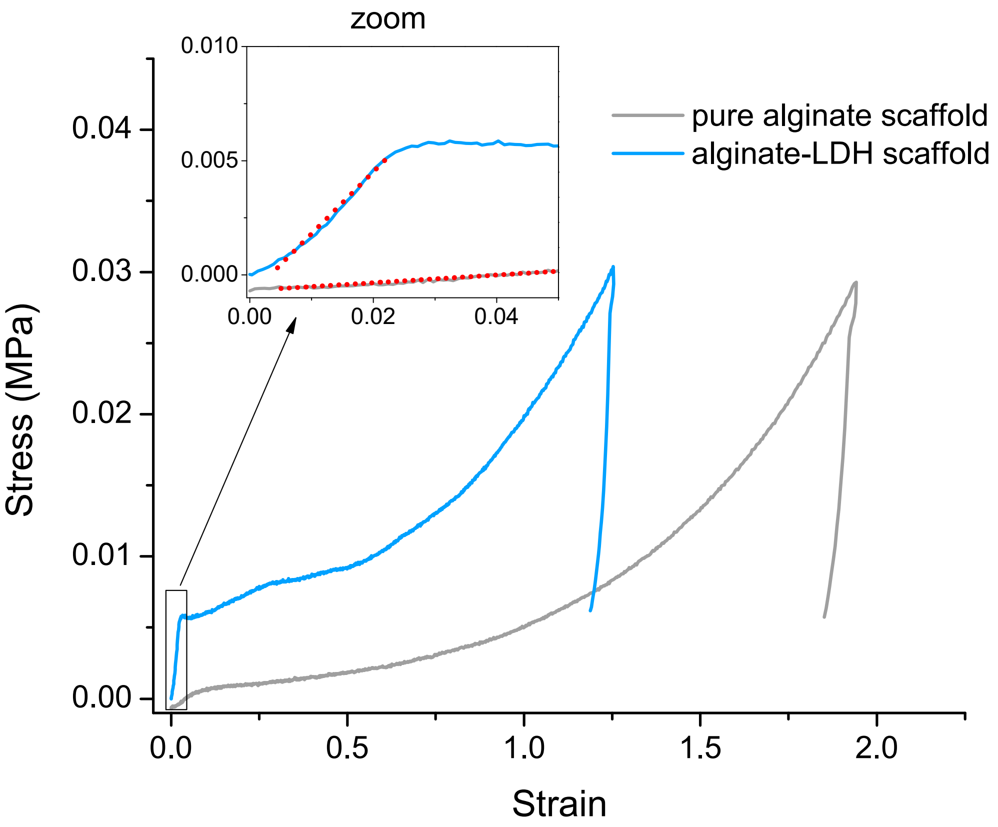

Figure S2. Evaluation of mechanical properties (stress-strain curves) through uniaxial compression for typical freeze-cast alginate/MgFeCO3 LDH and pure alginate scaffolds. The zoomed inlet view allows better visualizing the elastic part of the curves to decipher the variation in Young modulus (slope of the red dotted lines).

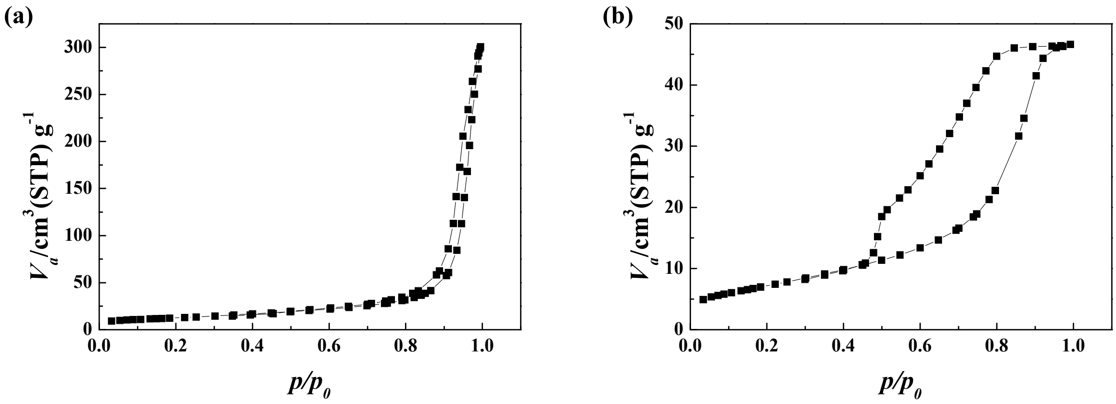

Figure S3. Evaluation of the porous volume by the BJH method, from processing nitrogen (N_2) adsorption/desorption data: (a) MgFeCO3 LDH powder, (b) SPS cold-sintered monolith.

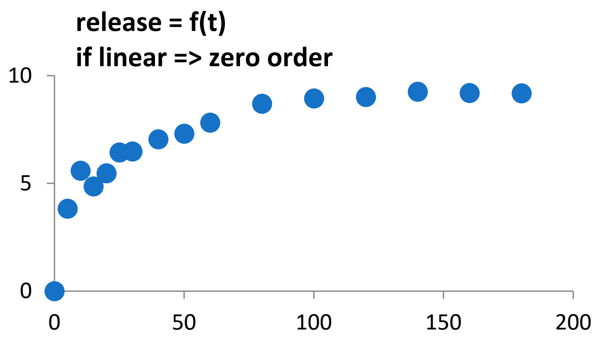
release = f(t)
if linear => zero order
10
5
0
0
50
100
150
200

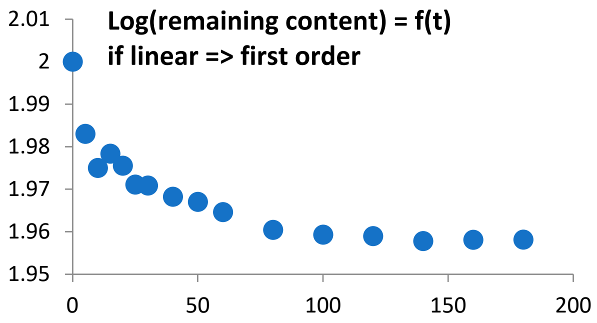
Log(remaining content) = f(t)
if linear => first order
2.01
2
1.99
1.98
1.97
1.96
1.95
0
50
100
150
200

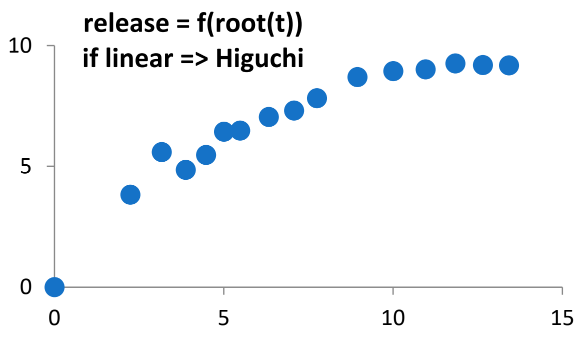
release = f(root(t))
if linear => Higuchi
10
5
0
0
5
10
15

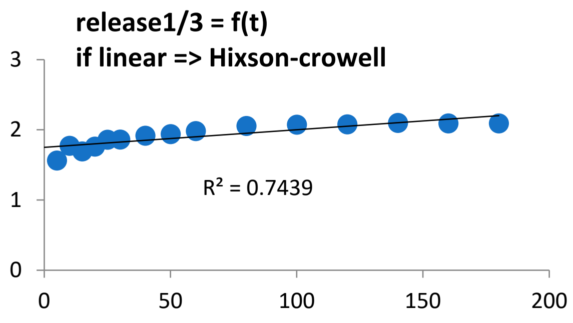
release1/3 = f(t)
if linear => Hixson-crowell
3
2
1
0
R² = 0.7439
0
50
100
150
200

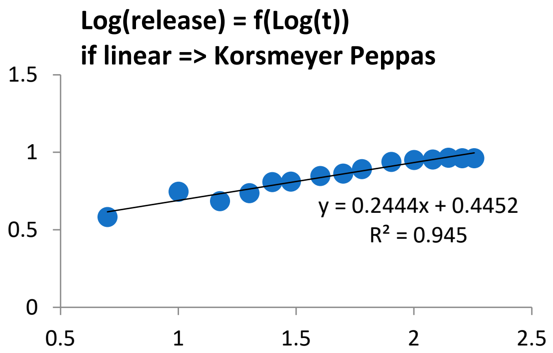

Figure S4. Mathematical fitting of MO release from $MgFeCO_3$ LDH monolith (from cold sintering) relatively to several models (t is given in minutes and the release rate in %).

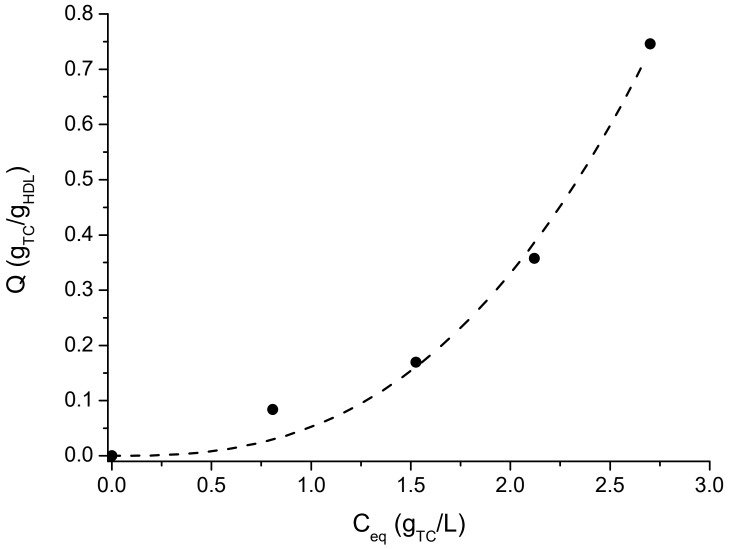

Figure S5. Isotherm of interaction between TC molecules and $MgFeCO_3$ LDH powder. The dotted line depicts the fit with Sips isotherm. Error bars are included but not visible (standard deviations correspond to 0.6% of the mean).

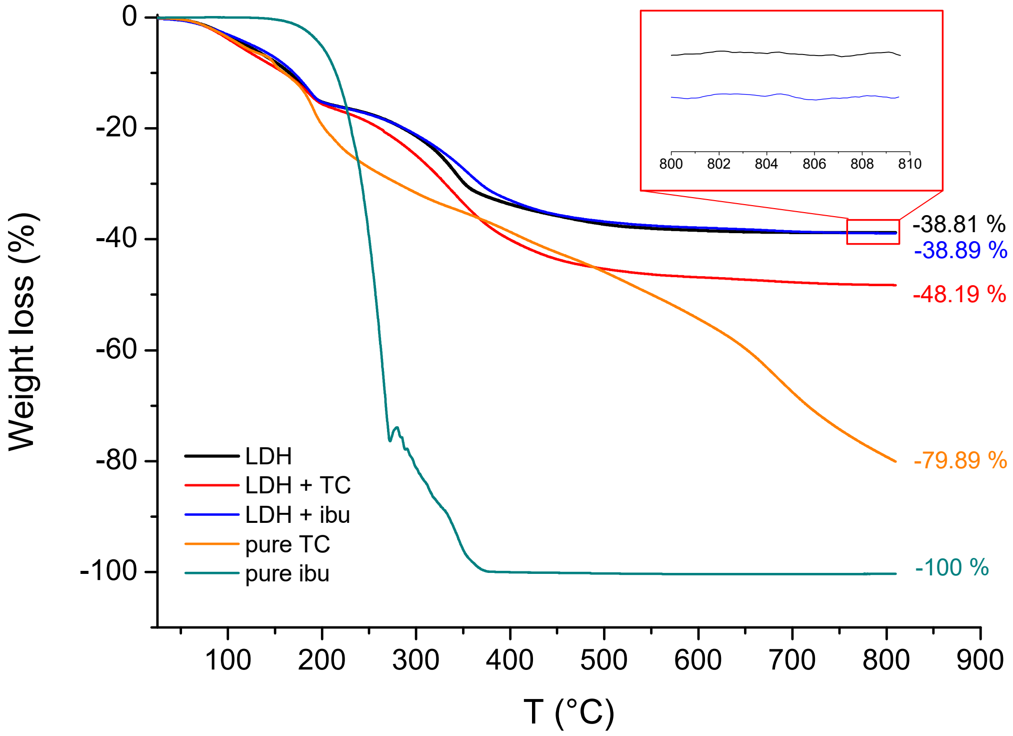

Figure S6. Thermogravimetry data on pure $MgFeCO_3$ LDH powder, tetracycline (TC), ibuprofene (ibu), and combined LDH-TC and LDH-ibu compounds.

Table S1. Correlation coefficient for the mathematical fit of the kinetics of adsorption of MO on $MgFeCO_3$ in powder or monoliths forms, using the pseudo-first order, pseudo-second order and Elovich kinetic models.

Sample	Kinetic model	R^2
MgFe-CO_3-LDH powder	Pseudo first order	0.9153
	Pseudo second order	0.9505
	Elovich	0.9689
MgFe-CO_3-LDH monolith	Pseudo first order	0.9742
	Pseudo second order	0.9895
	Elovich	0.9992
